# Identification of PDXDC1 as a novel pleiotropic susceptibility locus shared between lumbar spine bone mineral density and birth weight

**DOI:** 10.1007/s00109-021-02165-0

**Published:** 2022-03-22

**Authors:** Yu-Qian Song, Shi-Di Hu, Xu Lin, Xiang-He Meng, Xiao Wang, Yin-Hua Zhang, Cheng Peng, Rui Gong, Tao Xu, Tong Zhang, Chen-Zhong Li, Dao-Yan Pan, Jia-Yi Yang, Jonathan Greenbaum, Jie Shen, Hong-Wen Deng

**Affiliations:** 1 Department of Endocrinology and Metabolism, The Third Affiliated Hospital of Southern Medical University, Guangzhou, 510630 China; 2 Department of Endocrinology and Metabolism, Shunde Hospital of Southern Medical University, The First People’s Hospital of Shunde Foshan), Foshan, Guangdong China; 3 School of Basic Medical Science, Central South University, Changsha, Hunan 410013 People’s Republic of China; 4 Department of Geriatrics, School of Medicine, National Clinical Key Specialty, Guangzhou First People’s Hospital, South China University of Technology, Guangzhou, Guangdong China; 5 Cadre Ward Endocrinology Dept, Gansu Provincial Hospital, Lanzhou, Gansu, 730000 China; 6 Department of Epidemiology, College of Public Health, University of Iowa, Iowa City, IA USA; 7 Tulane Center for Biomedical Informatics and Genomics, Department of Medicine, Tulane University, New Orleans, LA USA

**Keywords:** Osteoporosis, Bone mineral density, Birth weight, Pleiotropy, Conditional false discovery rate

## Abstract

**Abstract:**

An increasing number of epidemiological studies have suggested that birth weight (BW) may be a determinant of bone health later in life, although the underlying genetic mechanism remains unclear. Here, we applied a pleiotropic conditional false discovery rate (cFDR) approach to the genome-wide association study (GWAS) summary statistics for lumbar spine bone mineral density (LS BMD) and BW, aiming to identify novel susceptibility variants shared between these two traits. We detected 5 novel potential pleiotropic loci which are located at or near 7 different genes (*NTAN1*, *PDXDC1*, *CACNA1G*, *JAG1*, *FAT1P1*, *CCDC170*, *ESR1*), among which *PDXDC1* and *FAT1P1* have not previously been linked to these phenotypes. To partially validate the findings, we demonstrated that the expression of *PDXDC1* was dramatically reduced in ovariectomized (OVX) mice in comparison with sham-operated (SHAM) mice in both the growth plate and trabecula bone. Furthermore, immunohistochemistry assay with serial sections showed that both osteoclasts and osteoblasts express *PDXDC1*, supporting its potential role in bone metabolism. In conclusion, our study provides insights into some shared genetic mechanisms for BMD and BW as well as a novel potential therapeutic target for the prevention of OP in the early stages of the disease development.

****Key messages**:**

- We investigated pleiotropy-informed enrichment between LS BMD and BW.
- We identified genetic variants related to both LS BMD and BW by utilizing a cFDR approach.
- PDXDC1 is a novel pleiotropic gene which may be related to both LS BMD and BW.
- Elevated expression of PDXDC1 is related to higher BMD and lower ratio n-6/n-3 PUFA indicating a bone protective effect of PDXDC1.

**Supplementary Information:**

The online version contains supplementary material available at 10.1007/s00109-021-02165-0.

## Introduction

The prevalence of osteoporosis (OP) in the USA was projected to increase by 17.2 million between 2010 and 2030 [1]. The lifetime fracture risk of patients with OP is estimated to be as high as 40% [2], and the economic burden associated with fractures is predicted to exceed $25 billion by 2050 without effective intervention of OP risk [3].

In recent decades, growing epidemiological evidence has shown that birth weight (BW) may be an important determinant of adult bone health. For instance, a previous study in British twins (*n* = 4008, 100% women) found that elevated BW has a protective effect on both bone mass and bone mineral content [4]. Similarly, research from The Gambia (*n* = 120, 57% men and 43% women) indicated that BW is positively correlated with the cross-sectional bone area at cortical sites in men and at trabecular sites in women [5]. Additionally, several studies have suggested that the intrauterine environment may program the embryogenesis genome with a skeletal growth trajectory that persists post-partum independent of post-natal factors [6, 7]. Given the close relationship between BW and bone health, and the high heritability of BW (87%) [8] and bone mineral density (BMD) (75–83%) [9], we hypothesized that there may be shared genetic determinants contributing to these two phenotypes.

Previous genome-wide association studies (GWAS) have identified at least 90 lumbar spine (LS) BMD-related loci and 87 BW-related loci, yet collectively these can only explain 12% and 15% of the individual trait variations, respectively [10, 11]. Here, we jointly analyzed GWAS summary statistics for BW and LS BMD using a pleiotropic conditional false discovery rate (cFDR) approach to identify novel trait-associated loci for the individual traits as well as those that may overlap [12]. The cFDR technique augments the effective sample size by efficiently combining existing datasets and has been successfully utilized by our team and others for exploring common pleiotropic loci for two related complex traits, such as coronary artery disease (CAD) and BMD [9], CAD and body mass index [13], or height and BMD [14].

The aim of this study was to identify potential novel pleiotropic susceptibility variants common to both BW and LS BMD using the cFDR method. Furthermore, to partially validate the reliability of the method and the genes identified by cFDR, we selected a novel pleiotropic gene to perform functional validation experiments in mice.

## Materials and methods

### GWAS datasets

BMD measured by dual-energy X-ray absorptiometry (DXA) is the gold standard for OP diagnosis as recommended by the WHO. Since LS BMD is estimated to have the highest heritability among commonly measured skeletal sites [15], it was thus chosen for studying the relationship between BMD and BW in this genetic association analysis.

The LS BMD GWAS summary-statistic dataset was acquired from the Genetic Factors for Osteoporosis Consortium (GEFOS) and included 53,236 individuals [16]. At the present time, it is the largest GWAS for DXA-derived BMD measurements. The BW summary statistics were downloaded from the Early Growth Genetics (EGG) Consortium and included 143,677 subjects [11]. To confirm that the variance estimated for each single-nucleotide polymorphism (SNP) was not inflated due to population stratification, standard genomic control procedures were applied to the two original GWAS studies. The two datasets have no overlapping subjects, and both include subjects of European ancestry.

### SNP pruning and merging

We performed linkage disequilibrium (LD)-based pruning for each dataset using PLINK version 1.9 software. Firstly, the LD was computed for each pair of SNPs in a window containing 50 SNPs. For pairs with an *r*^2^ > 0.2, the SNP with the smaller minor allele frequency was removed. The window was moved 5 SNPs forward, and the procedure was repeated until no pairs of SNPs across the genome had *r*^2^ > 0.2. The pruning was based on the LD structure of the CEU HapMap 3 genotypes. After the pruning process was completed, there were 121,848 SNPs which overlapped between BMD and BW which were retained for the subsequent analysis.

### Estimation of pleiotropic enrichment

Stratified quantile–quantile plots (Q-Q plots) were constructed to visualize the pleiotropic enrichment between LS BMD and BW when conditioning on successively more stringent *p* value thresholds of the conditional trait: *p* < 1 (all SNPs), *p* < 0.1, *p* < 0.01, and *p* < 0.001. The observed *p* values of the principal trait, denoted as “nominal -log_10_ (*p*),” were plotted on the *y*-axis against the empirical conditional *p* values, denoted as “empirical − log_10_ (q)”. The line *x* = *y* indicates the null hypothesis of no pleiotropic enrichment, and plots that deviate leftward from the null line indicate that a pleiotropic effect exists between the traits [12].

### Calculation of cFDR and ccFDR

The calculation of the cFDR extends from the single phenotype case, where the unconditional false discovery rate (FDR) for a set of SNPs is characterized as the probability of a false positive association. The cFDR expands this idea to the two-phenotype case and is defined as the probability of a false positive association with the principal trait given that the association *p*-values with both the principal and conditional traits are at least as small as the observed *p* values. Using the GWAS summary statistics, the cFDR for each SNP was separately computed for both orderings of the traits (BMD|BW and BW|BMD, where “|” indicates conditional upon). The SNPs were regarded as significantly related to the principal trait when cFDR < 0.05. More details are provided in Online Resource 1. The conjunction cFDR (ccFDR) value was defined as the maximum cFDR value of two trait orderings, and SNPs with ccFDR < 0.05 were interpreted as pleiotropic loci associated with both traits [12]. Manhattan plots were constructed using R to graphically display the genomic locations of significant variants.

### Annotation of novel SNPs and genes

The significant cFDR SNPs (cFDR < 0.05) were queried using the SNPinfo web server (https://snpinfo.niehs.nih.gov) to ascertain all corresponding SNPs with high LD (*r*^2^ > 0.8). All SNPs (including those with cFDR < 0.05 and those with high LD) were then compared to previous GWAS findings (*p* value < 5 × 10^−8^) on the European Bioinformatics Institute website. SNPs with a GWAS *p* value > 5 × 10^−8^ that have not been reported as having an association with BMD or/and BW were considered to be potential novel SNPs. We utilized both the SNP and CNV Annotation Database (SCAN, http://scandb.org/newinterface/about.html) and PubMed (https://www.ncbi.nlm.nih.gov/snp) to map the significant cFDR SNPs to nearby genes. Genes that were not previously identified in BMD or/and BW-related studies were deemed novel.

### Functional enrichment analysis and protein–protein interaction analysis of identified genes

To establish the physiological role of genes of interest, we performed functional enrichment analysis using the Database for Annotation, Visualization and Integrated Discovery (DAVID, https://david.ncifcrf.gov/summary.jsp). To explore the functional interactions between proteins produced by cFDR-significant pleiotropic susceptibility genes, we performed protein–protein interaction analysis by the online tool STRING 10.0 (http://string-db.org/).

### Fine-mapping

To discover putative pleiotropic causal SNPs and prioritize genes for the subsequent functional experiments, we performed multi-trait fine mapping analysis using PAINTOR [17]. In particular, we focused on a potential OP susceptibility locus located on chromosome 16 (14800000-16280000) which includes the gene *PDXDC1*.

### Postmenopausal OP mouse models

Female C57BL/6 mice were obtained from the Animal Center of Southern Medical University, which either received sham-operated (SHAM) surgery or ovariectomy (OVX) under 1.2% tribromoethanol anesthesia at 8 weeks of age. The OVX group had bilateral ovary removal, while the SHAM group had a similar volume of adipose tissue removed from around the ovaries. The mice were sacrificed sixteen weeks post-surgery and hind-limb specimens were harvested for subsequent analyses. Four mice were randomly selected from each experimental group.

### Micro-computed tomography analysis

The hindlimbs were fixed in 4% paraformaldehyde for 48 h then scanned in a micro-computed tomography (micro-CT) scanner (Viva CT40, Scanco Medical AG, Bassersdorf, Switzerland). Morphological analysis was conducted on trabecular bone. The region of interest in the trabecular bone began at a position 20 spongiosa slices (9 µm thick) from the lower growth plate of the femur and finished 160 slices later. Bone volume/total volume (BV/TV), trabecular thickness (Tb. Th), trabecular number (Tb. N), and trabecular separation (Tb. Sp) were computed using standard three-dimensional microstructural analysis.

### Immunohistochemistry

The hindlimbs were fixed in 4% paraformaldehyde in phosphate-buffered saline (PBS) for 48 h at 4 °C, then decalcified in 10% ethylenediaminetetraacetic acid (EDTA; pH 7.4) for 21 days at room temperature prior to dehydration in a rising gradient of ethanol and embedded in paraffin. The tissues were sliced into 3 or 4 micron thick sections for histological analysis. Tissue sections were incubated overnight at 4 °C with *PDXDC1* primary antibody (Proteintech, 21,021–1-AP, 1:200) then for 1 h at room temperature with a secondary antibody (Arigo, ARG65351, 1:200). Diaminobenzidine (DAB, KGP1045/KGP1045-20/KGP1045-100, 1:1:1:20) was conjugated to the secondary antibody.

Both *PDXDC1*-positive and the total number of cells were enumerated (at 400 × magnification) at the growth plate and within the trabecula bone in the femur or tibia. Four views from these regions picked at random were counted on each section, and three consecutive sections were selected for each mouse.

To distinguish whether the *PDXDC1*-positive cells were osteoclasts or osteoblasts, we conducted immunohistochemistry assay with three-micrometer-thick serial sections in the SHAM group. Tartrate-resistant acid phosphatase (TRAP) is a specific marker of osteoclasts and osteocalcin (OCN) is a specific marker of osteoblasts. The specific procedures of the immunohistochemistry assay with serial sections were as follows: (1) there were two thin and consecutive tissue sections on each glass slide labeled A and B in advance; (2) A-labeled sections were incubated with *PDXDC1* primary antibody (Proteintech, 21,021–1-AP, 1:200), and B-labeled sections were incubated with anti-OCN primary antibody (abcam, ab93876, 1:500) or subjected to TRAP staining (Sigma-Aldrich); (3) sections not used for TRAP staining were incubated with suitable secondary antibody (Arigo, ARG65351, 1:200).

### Fat-1 TG mouse model

To preliminarily verify the mechanism of *PDXDC1* affecting BW and BMD, a *fat-1* transgenic (TG) mouse model was adopted. Male *fat-1* TG mice were matched with wild-type (WT) female C57BL/6 mice to breed *fat-1* gene-positive mice. The *fat-1* gene-positive mice were identified using genomic DNA extracted from tail biopsies. Primer sequences were as follows: 5′-GGACCTGGTGAAGAGCATCCG-3′ and reverse, 5′-GCCGTCGCAGAAGCCAAAC-3′. We fed the mice until 16 months old when they were sacrificed and hind-limb specimens were harvested for subsequent analysis. We performed the immunohistochemical assay on both *fat-1* TG mice and WT mice with *PDXDC1* primary antibody (Proteintech, 21,021–1-AP, 1:200).

Sections were imaged using a Zeiss microscope (Carl Zeiss, New York, USA). All experiments were performed three times or more for reproducibility.

### Statistics

Data shown are means ± standard deviation (SD). Two sample *t*-tests were performed for comparison of experimental groups. Statistical significance was set at *p* value < 0.05.

### MR analysis

To investigate the potential causal relationship between BW and LS BMD, we performed two-sample Mendelian randomization (MR) analysis using the BW-associated SNPs as instrumental variables [18]. We first selected independent genetic variants (*r*^2^ ≤ 0.01) associated with BW (*p* value < 5 × 10^−8^) as the instrumental variables. We then obtained the corresponding effect estimates of these instrumental variable SNPs from both the BW and BMD GWAS analyses. The causal effects from multiple instruments were combined using several meta-analysis approaches including maximum likelihood estimation and inverse-variance weighting (IVW).

## Results

### Pleiotropy between LS BMD and BW

**Fig. 1:**
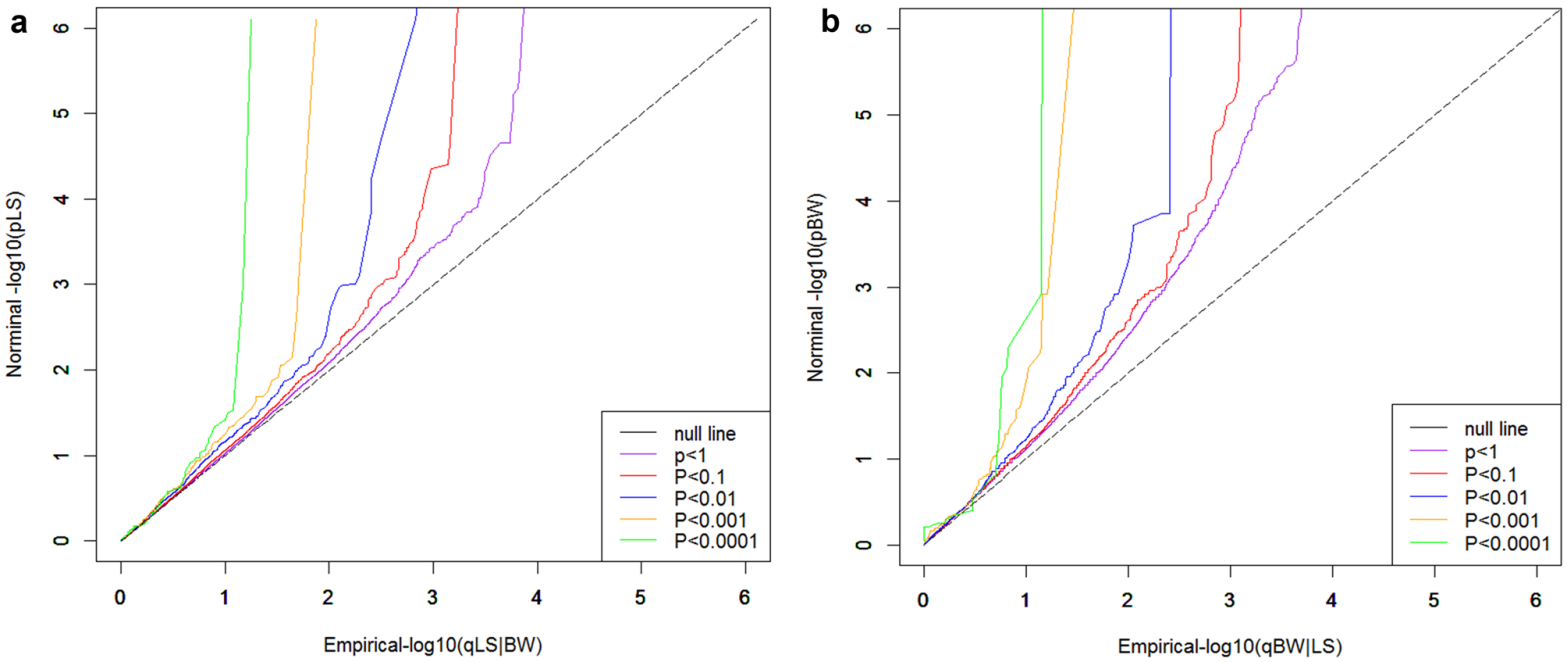
Q-Q plots. Stratified QQ plots of nominal versus empirical − log10 *p* values for **a** LS BMD as a function of significance of the association with BW, and **b** BW as a function of significance of the association with LS BMD. The level of − log10(*p*) > 0, − log10(*p*) > 1, − log10(*p*) > 2, − log10(*p*) > 3, − log10(*p*) > 4 correspond to *p* < 1, *p* < 0.1, *p* < 0.01, *p* < 0.001, *p* < 0.0001, respectively

The conditional Q-Q plots for LS BMD at various nominal *p* values of association with BW demonstrate enrichment over the null hypothesis line (i.e., leftward shift) at each stratified level of significance for BW (Fig. 1a), and vice versa for BW SNPs conditioned on LS BMD (Fig. 1b).

### LS BMD variants identified by cFDR

**Fig. 2:**
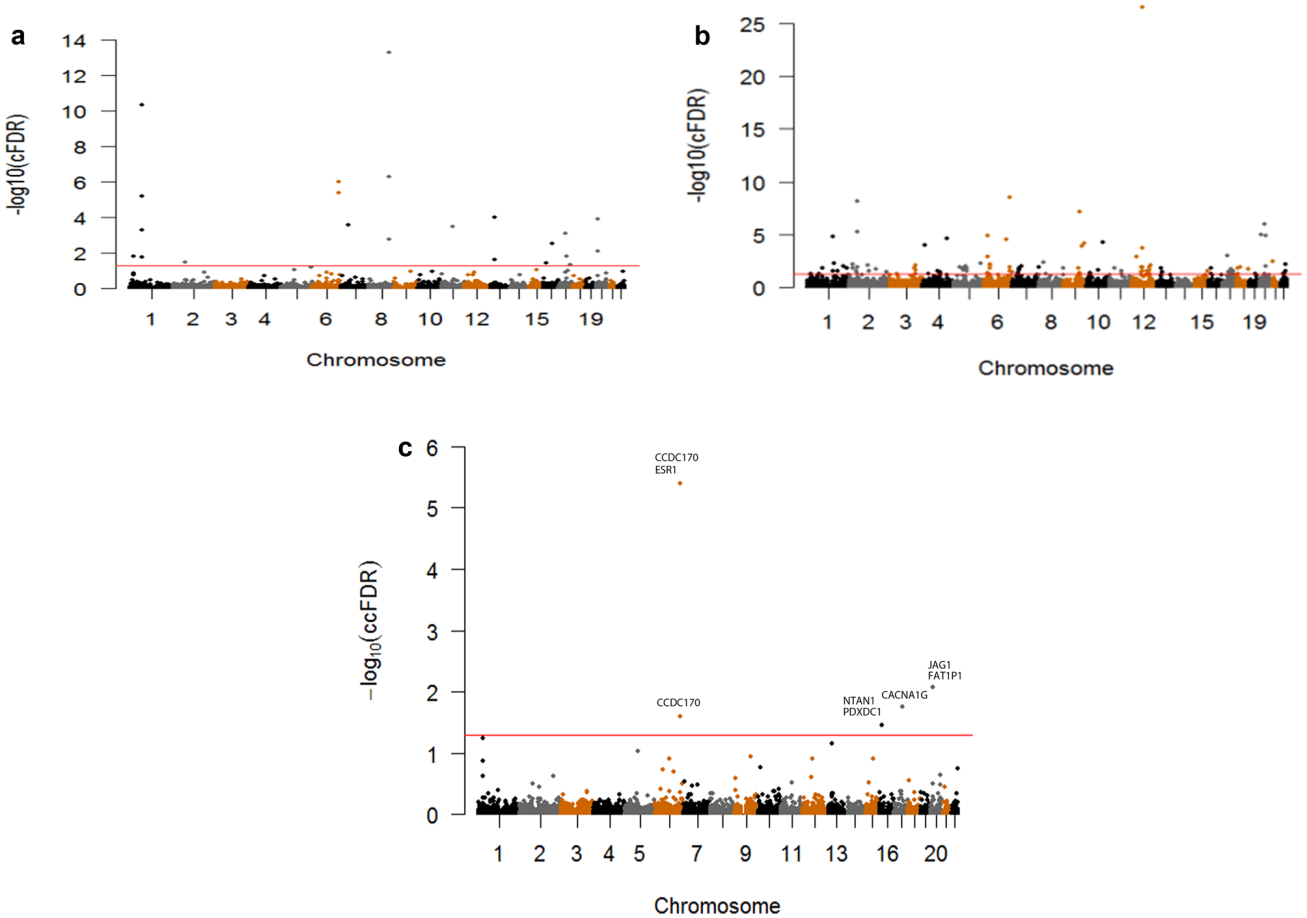
Manhattan plots. **a** Conditional Manhattan plot of conditional − log_10_ FDR values for LS BMD given BW (LS BMD|BW). **b** Conditional Manhattan plot of conditional − log_10_ FDR values for BW given LS BMD (BW|LS BMD). **c** Conjunction Manhattan plot of conjunction − log_10_ FDR values for LS BMD and BW. The red line corresponds to conditional − log_10_ FDR value of 1.3 (cFDR < 0.05)

Twenty-two LS BMD-associated SNPs conditioned on BW, mapping to 10 different chromosomes, were identified using the cFDR method (Fig. 2a). Among these 22 SNPs, 10 had a *p*-value less than 5 × 10^−8^ in the original LS BMD GWAS [16], and 1 SNP (*rs2741856*) was previously associated with BMD in another GWAS [19]. These 11 SNPs had therefore successfully replicated previous GWAS, partially demonstrating the reliability and robustness of the cFDR method. The remaining 11 novel SNPs with a *p* value > 5 × 10^−8^ would have easily been overlooked in a traditional single trait GWAS. The 22 SNPs were physically mapped to 27 different genes (Online Resource 2), 15 of which had previously been reported to be related to BMD or OP in various other studies[19–25]. In functional term enrichment analysis, some the identified variants were enriched in several terms associated with bone metabolism, such as “regulation of bone remodeling,” “regulation of bone resorption,” and “skeletal system development” (Table 1).

**Table 1:** Functional term enrichment analysis

GO term	Count	*p* value	FDR
LS BMD genes			
Regulation of bone remodeling	2	1.2E − 2	8.1E − 1
Regulation of bone resorption	2	1.2E − 2	8.1E − 1
Skeletal system development	3	2.6E − 2	8.4E − 1
BW genes			
Insulin-like growth factor receptor signaling pathway	5	5.1E − 7	4.9E − 4
Insulin receptor binding	5	1.4E − 5	1.9E − 3
Phosphatidylinositol 3-kinase signaling	4	3.0E − 4	9.2E − 2
Positive regulation of MAPK cascade	5	6.6E − 4	1.0E − 1
Protein binding	55	5.3E − 3	1.9E − 1
Regulation of growth	6	3.9E − 2	4.2E − 1

*GO term* gene ontology term

### BW variants identified by cFDR

Conditioned on the association with LS BMD, 99 BW-associated SNPs mapping to 20 different chromosomes (Fig. 2b), were identified as being associated with BW using the cFDR method. Among the 99 SNPs, 16 had a *p* value less than 5 × 10^−8^ in the original GWAS [11]. The remaining 83 SNPs with *p* values > 5 × 10^−8^, easily overlooked in traditional studies, were considered to be novel SNPs. The 99 SNPs were mapped to 130 different genes (Online Resource 3). Among these 130 different genes, 31 had been identified as having an association with BW in previous studies [9, 11, 26–32]. The functional term enrichment analysis results demonstrate that a considerable number of variants were enriched in terms such as “insulin-like growth factor receptor signaling pathway,” “insulin receptor binding,” “phosphatidylinositol 3-kinase signaling,” “positive regulation of MAPK cascade,” “protein binding,” and “regulation of growth” (Table 1). Notably, the abnormal expression and/or activation of IGF-1 receptor, phosphatidylinositol 3-kinase signal pathway, and MAPK family were confirmed to be associated with intrauterine growth restriction of placentas [33]. Furthermore, vitamin D-binding protein, adipocyte fatty acid-binding protein, and insulin-like growth factors binding proteins have been reported to have a key role in modulating BW [34–36].

### Pleiotropic variants for both LS BMD and BW identified by ccFDR

We identified 5 pleiotropic susceptibility SNPs (ccFDR < 0.05) associated with both LS BMD and BW (*rs34955778*, *rs198542*, *rs2423512*, *rs12197879*, *rs1293935*), which mapped to 4 different chromosomes and 7 different genes (Fig. 2c, Table 2). Among these genes, *CACNA1G*, *JAG1*, and *ESR1* were previously reported to be related to both BMD [20, 23] and BW [9, 11], while *CCDC170* and *NTAN1* were previously reported to have an association with BMD [20, 24]. The remaining 2 genes (*PDXDC1* and *FAT1P1*) have not previously been identified to be associated with either trait.

**Table 2:** Pleiotropic SNPs for both LS BMD and BW

	SNP	Nearby gene	CHR	cFDR.LS	cFDR.BW	ccFDR
1	rs12197879	CCDC170	6	1.01E − 06	2.55E − 02	2.55E − 02
2	rs1293935	CCDC170	6	3.95E − 06	2.80E − 09	3.95E − 06
		ESR1				
3	rs34955778	NTAN1	16	3.59E − 02	1.60E − 02	3.59E − 02
		PDXDC1				
4	rs198542	CACNA1G	17	1.49E − 02	1.74E − 02	1.74E − 02
5	rs2423512	JAG1	20	8.36E − 03	9.41E − 06	8.36E − 03
		FAT1P1				

*SNP* single nucleotide polymorphisms, *CHR* chromosome, *cFDR.LS* conditional false discovery rate of LS BMD when conditioned on BW, *cFDR.BW* conditional false discovery rate of BW when conditioned on LS BMD, *ccFDR* conjunction conditional false discovery rate

**Fig. 3:**
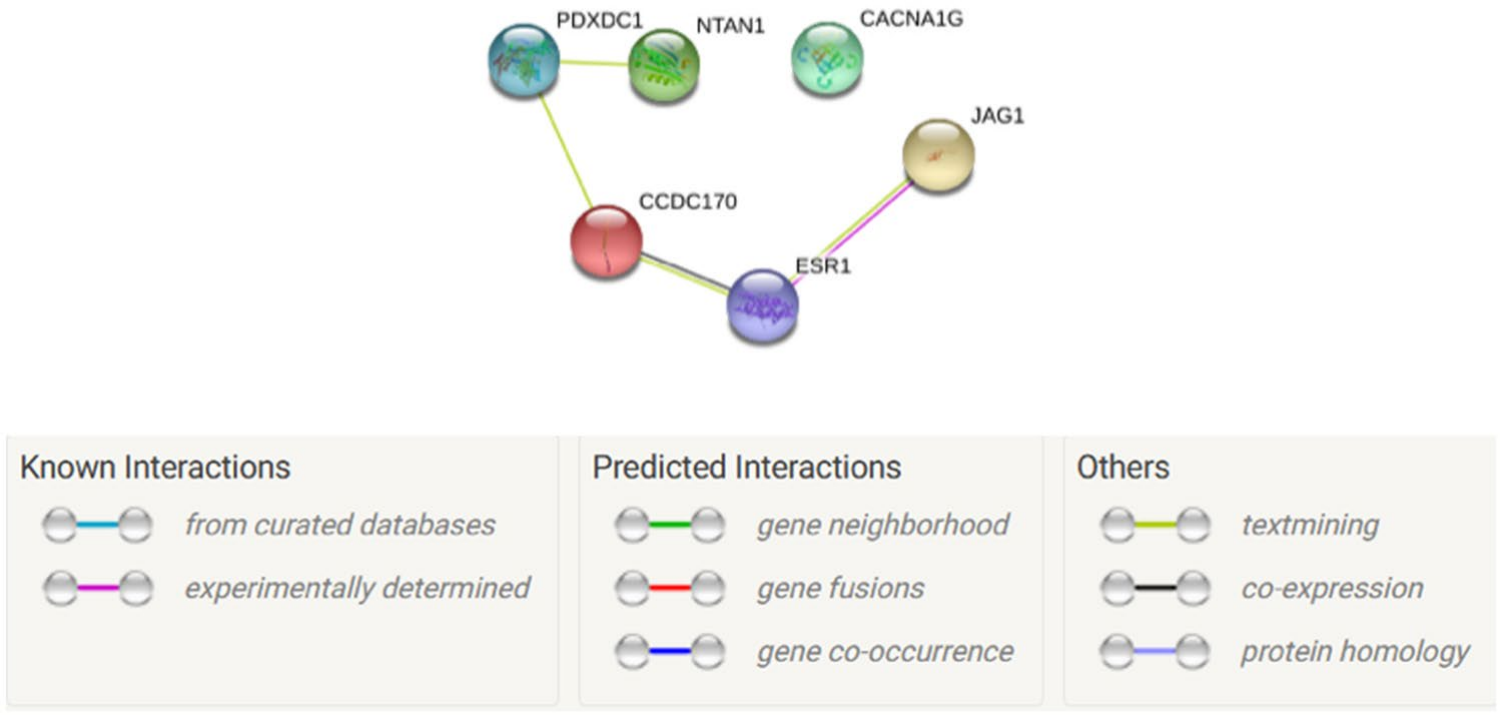
Protein–protein interaction network. Protein–protein interaction network for cFDR-significant pleiotropic susceptibility genes. Connections were based on evidence with an interaction score ≥ 0.40. Network nodes represent proteins produced by the corresponding genes, edges between nodes indicate protein–protein associations, edge color indicates the type of interaction and is specified on the bottom of the figure

*PDXDC1* was connected with *CCDC170* and *NTAN1* in the protein–protein interaction network constructed for the cFDR-significant pleiotropic susceptibility genes (Fig. 3), hinting at a potential role for *PDXDC1* in bone health [20].

### Fine-mapping

**Fig. 4:**
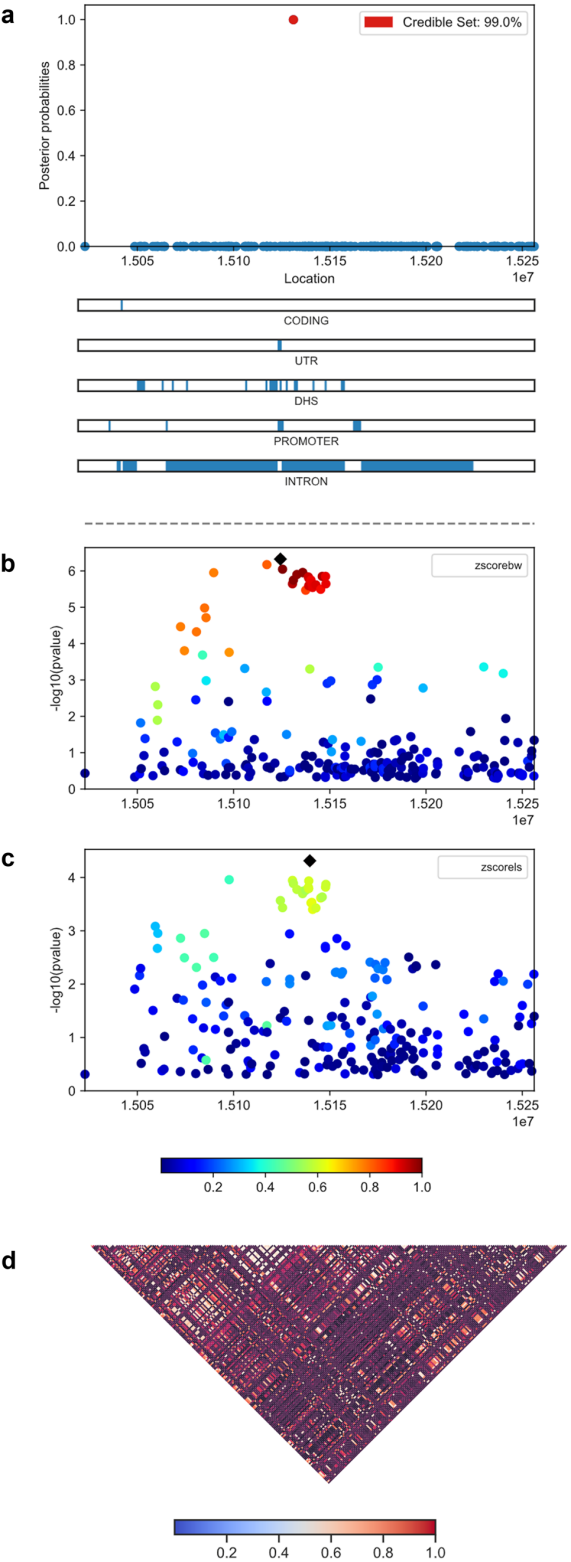
Visualization of posterior probability plots. We present **a** posterior probability of each SNP, **b** Z-score for BW, **c** Z-score for LS BMD, and **d** LD matrix

Based on the multi-trait fine mapping analysis, the SNP *rs1121*, which is located in the intron region of *PDXDC1*, was identified to have the highest posterior probability of causality (0.99) for both traits (Fig. 4). Additionally, this putative pleiotropic causal SNP is an expression quantitative trait loci (eQTL) associated with *PDXDC1* expression in muscle skeletal tissue (*p* value = 9.58 × 10^7^) in the GTEx database (https://www.gtexportal.org/home/index.html). Furthermore, *PDXDC1* has been linked with both omega-3 (n-3) and omega-6 (n-6) polyunsaturated fatty acids (PUFAs) in previous GWAS [37, 38]. We note that n-3 and n-6 PUFAs were demonstrated to be critical for both fetal growth and the regulation of bone metabolism [39, 40]. It was reported that elevated n-6/n-3 PUFA ratio is related to lower BMD of hip [41]. Based on these findings, we hypothesized that *PDXDC1* might causally influence BW and BMD through the modulation of PUFAs levels.

### Partial validation based on animal models

**Fig. 5:**
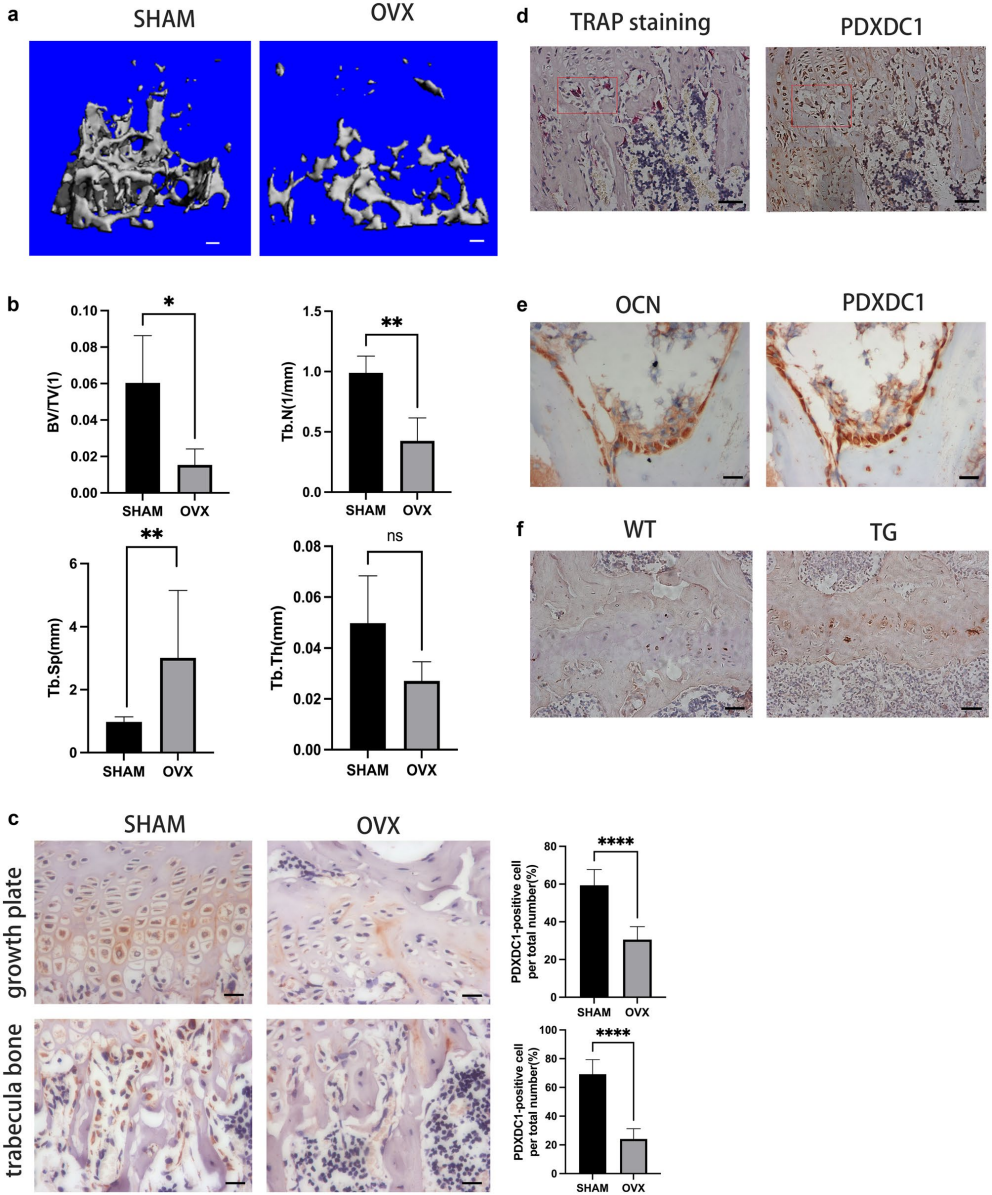
Partially validation of *PDXDC1*. **a** Micro-computed tomography scanner assessment of trabecular bone microstructural parameters. Three-dimensional microstructural of trabecular bone of SHAM group and OVX group by micro-CT scanner at 16 weeks after operation. Scale bar, 100 μm. **b** Morphological analysis between SHAM group and OVX group of BV/TV, Tb. Th, Tb. N, and Tb. Sp. *n* = 4 per group. Data represent mean ± SD. *p < 0.05, **p < 0.01. **c** Immunostaining and quantification analysis of *PDXDC1* between SHAM group and OVX group at growth plate and trabecula bone. Higher expression of *PDXDC1* is related to higher BMD. Scale bar, 20 μm. *n* = 4 per group. Data represent mean ± SD. ****p < 0.0001. **d**, **e** Immunohistochemistry assay with serial sections of SHAM group. Both osteoclasts and osteoblasts express *PDXDC1*. Scale bar of **d**, 50 μm. Scale bar of **e**, 20 μm. **f** Immunostaining of *PDXDC1* of transgenic (TG) mice and wild-type (WT) mice. Higher expression of *PDXDC1* is related to lower ratio n-6/n-3 PUFA. Scale bar, 50 μm

*PDXDC1* was selected as the target gene to test for its association with LS BMD by immunohistochemical analysis for its protein levels in a postmenopausal OP mouse model to partially verify the validity of the cFDR method. Sixteen weeks after ovariectomy, morphometry analysis of trabecular bone demonstrated significantly lower BV/TV (*p* value < 0.05), less Tb. N (*p* value < 0.01), and wider Tb. Sp (*p* value < 0.01) in the OVX group compared to the SHAM group. Tb. Th between the two groups was not significantly different (Fig. 5a, b). The immunohistochemical assay showed that quantities of *PDXDC1*-positive cells were present within the growth plate and on the surface of trabecula bone. The expression of *PDXDC1* in the growth plate and trabecula bone of the OVX group decreased dramatically compared to the SHAM group (*p* value < 0.0001) (Fig. 5c). The immunohistochemistry assay with serial sections showed that both osteoclasts and osteoblasts expressed *PDXDC1* (Fig. 5d, e).

**Fig. 6:**
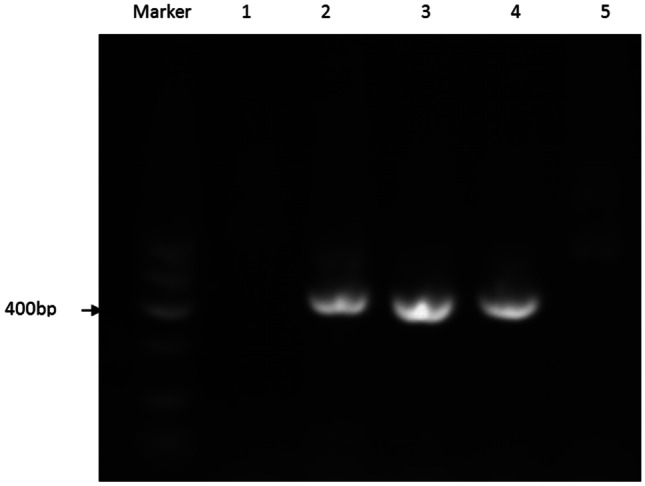
*fat-1* TG mice identification. PCR genotyping identification with *fat-1* fragment-specific primers. Lines 2, 3, and 4 indicated *fat-1* gene expression; lines 1 and 5 indicated wild-type (WT) gene expression

To preliminarily verify the mechanism of *PDXDC1* affecting BW and BMD, we performed the immunohistochemical assay on *fat-1* TG mice and WT mice. The *fat-1* TG mice can convert n-6 to n-3 PUFAs endogenously and have been shown to display elevated n-3 PUFAs and lower n-6 PUFAs in both cartilage and serum [42]. We performed PCR genotyping identification with *fat-1* fragment-specific primers to identify the *fat-1* TG mice (Fig. 6). The immunohistochemical assay results showed that the expression of *PDXDC1* in the growth plate of *fat-1* TG mice was increased dramatically compared to WT mice (Fig. 5f). This suggests that *PDXDC1* may have a protective role for BMD by interacting with n-3 and n-6 PUFAs.

### Two-sample MR analysis

We obtained 46 independent SNPs that associated with BW which were included in the MR analysis. However, we were not able to detect a significant causal effect of BW on BMD (Online Resource 4).

## Discussion

In this study, we investigated pleiotropy-informed enrichment between LS BMD and BW and identified genetic variants related to both traits by utilizing a cFDR approach. With a cFDR-significance threshold of 0.05, we identified 22 SNPs associated with LS BMD, including 11 novel loci overlooked in previous GWAS [16, 19], 99 SNPs associated with BW, including 83 novel loci ignored in the original GWAS [11], and 5 novel pleiotropic loci which may be related to both LS BMD and BW.

Interestingly, since the variants identified as being associated with either LS BMD or BW by cFDR were conditioned on one another, several may demonstrate pleiotropic effects. For example, *HMGA2*, identified to be associated with BW in this study in addition to other research [43], has also been reported to influence trabecular BMD in elderly men [44]. Additionally, the expression of *HMGA2* has been detected in the human fetal osteoblast cell line hFOB [44]. *WNT4*, identified as being associated with LS BMD by cFDR, was previously reported to be associated with BW in a large-scale GWAS [11]. *IGF-1*, identified to be associated with BW by cFDR, was established to regulate BMD in women in a population-based study [45]. It has been proposed that that *IGF-1* may be modulated by changes in estrogen levels, which in turn affects BMD [45]. Lastly, *RB1*, identified to be associated with BW in this study, has been confirmed to be related to mineralization defects in bones in a previous animal study [46].

The 5 identified pleiotropic susceptibility loci for both LS BMD and BW were mapped to 7 different genes, among which *PDXDC1* and *FAT1P1* not previously been linked to these phenotypes are particularly interesting. *PDXDC1*, preferentially expressed in the intestine, encodes the protein vitamin B6-dependent decarboxylase [47]. This gene has been reported to have an association with both n-3 and n-6 PUFAs [37, 38]. We demonstrated that the expression of *PDXDC1* in OVX mice was significantly reduced compared to SHAM group mice. Additionally, the expression of *PDXDC1* in *fat-1* TG mice increased dramatically compared to WT mice. The findings hint that elevated expression of *PDXDC1* is related to higher BMD and lower ratio n-6/n-3 PUFA. We also successfully verified that both osteoclasts and osteoblasts express *PDXDC1*. These results are in accordance with previous studies in both humans and mice which reported that lower n-6/n-3 PUFA ratio is beneficial for bone health [41, 48]. Both n-3 and n-6 PUFAs have been shown to modulate the function of osteoclasts and osteoblasts through cell adhesion [40]. Furthermore, n-3 PUFAs of maternal blood were found to be positively correlated with BW by reducing blood viscosity and increasing placental blood flow [49]. In future follow-up studies, we may develop *PDXDC1* knockout mice to explore the molecular mechanisms of how *PDXDC1* interacting with n-3 and n-6 PUFAs regulate BW and BMD concurrently. *FAT1P1* is a pseudogene of *FAT1*, and we know limitedly.

Although we successfully applied the cFDR methodology to identify novel variants associated with BW and/or BMD, there are several limitations to the current research. Firstly, we cannot assess the proportion of variability in the phenotypic traits explained by the identified loci since we could not obtain the individual level genotype data. Secondly, we were not able to distinguish between the pleiotropic scenarios where either the variant separately influences both traits or the variant influences BW which in turn influences BMD. Thirdly, our research did not verify the molecular mechanisms of *PDXDC1* concurrently regulating BW and BMD. However, theoretical and biological investigation may be pursued in future studies to reveal the common pathophysiological mechanisms of both traits.

In conclusion, our study indicates that *PDXDC1* may benefit to bone health and provides insights into some shared genetic mechanisms for BMD and BW as well as a novel potential therapeutic target for the prevention of OP in the early stages of the disease development.

## Supplementary Information

Below is the link to the electronic supplementary material.

Supplementary file1 (PDF 280 KB)

Supplementary file2 (PDF 100 KB)

Supplementary file3 (PDF 125 KB)

Supplementary file4 (PDF 77 KB)

Supplementary file5 (TIF 4643 KB)

## Data Availability

The authors confirm that the data and material supporting the findings of this study are available within the article and its supplementary materials.
