# Supplementary material for: Identification of PDXDC1 as a novel pleiotropic susceptibility locus shared between lumbar spine bone mineral density and birth weight
Source: J Mol Med (Berl). 2022 Mar 22;100(5):723–34. doi: 10.1007/s00109-021-02165-0 (PMC9110509; doi:10.1007/s00109-021-02165-0)
Supplement: Supplementary file 1 — Supplementary file1 (PDF 280 KB) [file 109_2021_2165_MOESM1_ESM.pdf]

Identification of Potential Novel Pleiotropic Susceptibility Variants Common to Lumbar Spine Bone Mineral Density and Birth Weight

Yu-Qian Song, Shi-Di Hu, Xu Lin, Xiang-He Meng, Xiao Wang, Yin-Hua Zhang, Cheng Peng, Rui Gong, Tao Xu, Tong Zhang, Chen-Zhong Li, Dao-Yan Pan, Jia-Yi Yang, Jonathan Greenbaum, Jie Shen*, Hong-Wen Deng*

Corresponding authors:

Hong-wen Deng, Ph. D.

Tulane Center for Biomedical Informatics and Genomics, School of Medicine, Tulane University, New Orleans, LA 70112, USA

School of Basic Medical Science, Central South University, Changsha, Hunan 410013, P. R., China

Tel: 1 504-988-1310 E-mail: hdeng2@tulane.edu

Jie Shen, Ph. D.

Department of Endocrinology and Metabolism, The Third Affiliated Hospital of Southern Medical University, Guangzhou, China.

Department of Endocrinology and Metabolism, Shunde Hospital of Southern Medical University (The First People's Hospital of Shunde Foshan), Foshan, Guangdong, China

Email: sjiesy@smu.edu.cn

Journal name: Journal of Molecular Medicine

Supplementary Materials

Calculation of cFDR

$$\text{cFDR}(p_i|p_j) = \Pr\,(H_0^{(i)}|P_i \le p_i, P_j \le p_j)$$

The cFDR is expressed as cFDR($p_i|p_j$). The p_i and p_j represent the observed strength of association for a particular variant with the principal phenotype and the conditional phenotype respectively. $H_0^{(i)}$ represents the null hypothesis that a particular SNP is not associated with the principal trait.

Supplementary phenotypes

We acquired both femoral neck (FN) BMD and BMD estimated by quantitative ultrasound of the heel (eBMD) GWAS summary-statistics datasets from the Genetic Factors for Osteoporosis Consortium (GEFOS) and UK Biobank and performed cFDR conditional upon BW respectively.

The stratified Q-Q plots for FN BMD conditioned on BW and for BW conditioned on FN BMD were both observed to have clear separation between the different stratification groups, demonstrating pleiotropic enrichment between those two traits **(Supplementary Fig. 1a, b**).

Similarly, the stratified Q-Q plots for eBMD conditioned on BW and BW conditioned on eBMD were both observed to have clear separation between the different levels, demonstrating pleiotropic enrichment between those two traits **(Supplementary Fig.1c, d)**.

We identified 2 pleiotropic susceptibility SNPs (rs12197879 and rs1293935) shared between FN BMD and BW **(Supplementary Table 1)**, LS BMD and BW, as well as eBMD and BW. We identified 51 pleiotropic susceptibility SNPs (ccFDR < 0.05) associated with both eBMD and BW **(Supplementary Table 2)**.

Supplementary table and figure tables

Supplementary Table 1. Pleiotropic SNPs for both FN BMD and BW

Supplementary Table 2. Pleiotropic SNPs for both eBMD and BW

Supplementary Fig. 1 Q-Q plots Stratified QQ plots of nominal versus empirical -log10 *p*-values for **(a)** FN BMD as a function of significance of the association with BW, and **(b)** BW as a function of significance of the association with FN BMD. Stratified QQ plots of nominal versus empirical -log10 *p*-values for **(c)** eBMD as a function of significance of the association with BW, and **(d)** BW as a function of significance of the association with eBMD. The level of -log10(p) > 0, -log10(p) > 1, -log10(p) > 2, -log10(p) > 3, -log10(p) > 4 correspond to $p < 1$, $p < 0.1$, $p < 0.01$, $p < 0.001$, $p < 0.0001$ respectively.

Supplementary Table 1. Pleiotropic SNPs for both FN BMD and BW

	SNP	Nearby Gene	CHR	cFDR.FN	cFDR.BW	ccFDR
1	rs12197879	CCDC170	6	3.38E-07	1.53E-02	1.53E-02
2	rs1293935	CCDC170<br>ESR1	6	1.86E-04	7.20E-09	1.86E-04

Column definition: SNP, single nucleotide polymorphisms; CHR, chromosome; cFDR.FN, conditional false discovery rate of FN BMD when conditioned on BW; cFDR.BW, conditional false discovery rate of BW when conditioned on FN BMD; ccFDR, conjunction conditional false discovery rate.

Supplementary Table 2. Pleiotropic SNPs for both eBMD and BW

	SNP	Nearby Gene	CHR	cFDR.eBMD	cFDR.BW	ccFDR
1	rs1766807	LOC107985447	1	2.74E-09	4.20E-02	4.20E-02
2	rs12026324	TBX15	1	2.70E-06	9.98E-03	9.98E-03
3	rs7549007	TCHHL1	1	4.67E-03	3.29E-03	4.67E-03
4	rs10753804	LOC284688	1	7.98E-06	1.05E-02	1.05E-02
5	rs1415181	LOC100129402<br>LOC100505982<br>GAPDHP24	1	3.70E-03	5.59E-04	3.70E-03
6	rs7547731	HSPG2	1	1.88E-02	2.37E-02	2.37E-02
7	rs7544210	WNT4	1	1.09E-02	1.93E-03	1.09E-02
8	rs4655036	WNT4	1	8.08E-13	3.92E-02	3.92E-02
9	rs1473488	TGFBR3	1	1.24E-02	1.37E-02	1.37E-02
10	rs7632	KLF11	2	2.89E-03	2.03E-02	2.03E-02
11	rs4675095	IRS1	2	3.51E-02	5.88E-03	3.51E-02
12	rs1451156	PIGF	2	4.10E-03	6.34E-03	6.34E-03
13	rs10168767	LOC102724072	2	6.16E-59	4.90E-03	4.90E-03
14	rs7612543	ZBTB38	3	4.17E-02	2.93E-02	4.17E-02
15	rs10049090	LINC02029	3	2.50E-17	8.27E-39	2.50E-17
16	rs10461018	CCDC12	3	9.29E-03	1.90E-03	9.29E-03
17	rs12643660	PPA2	4	2.00E-03	4.65E-03	4.65E-03

18	rs28673203	ANAPC10	4	6.37E-05	1.48E-02	1.48E-02
19	rs740672	FAM184B	4	1.28E-03	3.83E-06	1.28E-03
20	rs6853216	LCORL	4	9.00E-07	1.30E-08	9.00E-07
21	rs3849774	DAB2	5	4.10E-06	1.06E-02	1.06E-02
22	rs1975427	LHFPL2	5	3.74E-02	2.07E-02	3.74E-02
23	rs10457487	RSPO3	6	3.15E-130	3.30E-04	3.30E-04
		LOC112267972				
24	rs1415701	L3MBTL3	6	1.98E-13	1.06E-07	1.06E-07
25	rs7757002	AKAP12	6	2.29E-02	1.97E-02	2.29E-02
26	rs12197879	CCDC170	6	1.46E-122	1.02E-02	1.02E-02
27	rs1293935	CCDC170	6	1.20E-91	1.20E-09	1.20E-09
		ESR1				
28	rs1361024	ESR1	6	1.49E-06	1.15E-03	1.15E-03
29	rs9371554	ESR1	6	1.11E-02	3.98E-02	3.98E-02
		LOC107986529				
30	rs2347902	CALD1	7	4.15E-02	2.65E-02	4.15E-02
31	rs7460241	GPR20	8	4.94E-03	1.08E-02	1.08E-02
32	rs9409609	ROR2	9	3.59E-02	4.26E-02	4.26E-02
33	rs357542	FANCC	9	4.39E-04	1.44E-02	1.44E-02
34	rs35532379	PTCH1	9	1.76E-03	4.12E-02	4.12E-02
35	rs28536742	PTCH1	9	1.62E-11	1.22E-09	1.22E-09

36	rs10764396	C10orf67	10	9.01E-03	1.88E-02	1.88E-02
37	rs6480933	LOC105378305	10	2.06E-100	1.03E-02	1.03E-02
38	rs1949095	LOC105378305	10	5.48E-46	1.21E-02	1.21E-02
39	rs10773000	MTRFR	12	2.93E-03	7.72E-03	7.72E-03
40	rs12828089	SLC38A1	12	1.93E-02	4.61E-03	1.93E-02
41	rs1042725	HMGA2	12	2.00E-03	3.78E-29	2.00E-03
42	rs11175992	LINC02425	12	9.10E-06	2.17E-04	2.17E-04
43	rs772313	DOCK9	13	4.32E-10	2.21E-03	2.21E-03
44	rs1951868	BMP4	14	1.90E-04	4.35E-02	4.35E-02
45	rs16950543	LOC102723493	15	2.81E-02	2.37E-02	2.81E-02
46	rs11631839	SMAD3	15	7.71E-05	4.77E-02	4.77E-02
47	rs4783585	FHOD1	16	2.36E-03	4.63E-02	4.63E-02
48	rs9938631	ZDHHC1	16	1.43E-05	2.76E-02	2.76E-02
49	rs2052412	ZDHHC1	17	2.73E-03	2.34E-02	2.34E-02
50	rs757558	AXIN2	17	6.30E-03	2.67E-03	6.30E-03
51	rs9962540	CABLES1	18	2.81E-02	1.36E-03	2.81E-02

Column definition: SNP, single nucleotide polymorphisms; CHR, chromosome; cFDR.eBMD, conditional false discovery rate of eBMD when conditioned on BW; cFDR.BW, conditional false discovery rate of BW when conditioned on eBMD; ccFDR, conjunction conditional false discovery rate.

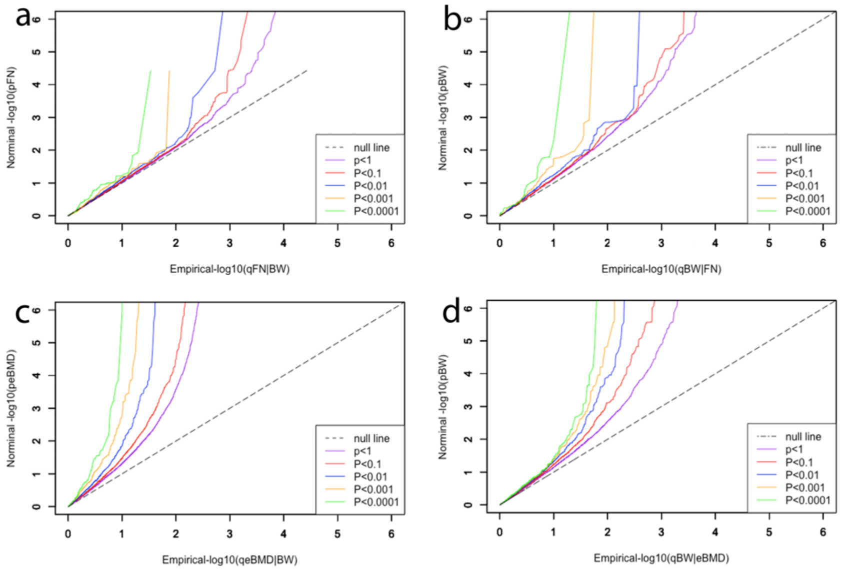

Supplementary Fig. 1 Q-Q plots Stratified QQ plots of nominal versus empirical -log10 *p*-values for **(a)** FN BMD as a function of significance of the association with BW, and **(b)** BW as a function of significance of the association with FN BMD. Stratified QQ plots of nominal versus empirical -log10 *p*-values for **(c)** eBMD as a function of significance of the association with BW, and **(d)** BW as a function of significance of the association with eBMD. The level of -log10(p) > 0, -log10(p) > 1, -log10(p) > 2, -log10(p) > 3, -log10(p) > 4 correspond to $p < 1$, $p < 0.1$, $p < 0.01$, $p < 0.001$, $p < 0.0001$ respectively.
