# Supplementary material for: Identification of PDXDC1 as a novel pleiotropic susceptibility locus shared between lumbar spine bone mineral density and birth weight
Source: J Mol Med (Berl). 2022 Mar 22;100(5):723–34. doi: 10.1007/s00109-021-02165-0 (PMC9110509; doi:10.1007/s00109-021-02165-0)
Supplement: Supplementary file 2 — Supplementary file2 (PDF 100 KB) [file 109_2021_2165_MOESM2_ESM.pdf]

Identification of Potential Novel Pleiotropic Susceptibility Variants Common to Lumbar Spine Bone Mineral Density and Birth Weight

Yu-Qian Song, Shi-Di Hu, Xu Lin, Xiang-He Meng, Xiao Wang, Yin-Hua Zhang, Cheng Peng, Rui Gong, Tao Xu, Tong Zhang, Chen-Zhong Li, Dao-Yan Pan, Jia-Yi Yang, Jonathan Greenbaum, Jie Shen*, Hong-Wen Deng*

*** Corresponding authors:**

Hong-wen Deng, Ph. D.

Tulane Center for Biomedical Informatics and Genomics, School of Medicine, Tulane University, New Orleans, LA 70112, USA

School of Basic Medical Science, Central South University, Changsha, Hunan 410013, P. R., China

Tel: 1 504-988-1310 E-mail: hdeng2@tulane.edu

Jie Shen, Ph. D.

Department of Endocrinology and Metabolism, The Third Affiliated Hospital of Southern Medical University, Guangzhou, China.

Department of Endocrinology and Metabolism, Shunde Hospital of Southern Medical University (The First People's Hospital of Shunde Foshan), Foshan, Guangdong, China

Email: sjiesy@smu.edu.cn

Journal name: Journal of Molecular Medicine

Online Resource 2. LS BMD SNPs given BW (cFDR<0.05)

	SNP	CHR	Nearby Gene	*p*.value LS	cFDR.LS
1	rs2473252	1	WNT4[a]	2.18E-05	1.47E-02
			LOC343384[c]		
2	rs1864569	1	WLS[b]	2.80E-06	1.81E-02
3	rs2566758	1	WLS[b]	4.07E-08	4.99E-04
4	rs944082	1	WLS[b]	3.36E-10	6.70E-06
5	rs7554551	1	WLS[b]	1.29E-15	4.71E-11
6	rs4854512	2	ARHGAP25[b]	5.06E-06	3.45E-02
7	rs12197879	6	CCDC170[b]	7.31E-10	1.01E-06
8	rs1293935	6	CCDC170[b]	7.89E-07	3.95E-06
			ESR1[a]		
9	rs4346915	7	EPDR1[b]	1.92E-08	2.71E-04
			STARD3NL[b]		
10	rs2055101	8	LOC441377[c]	9.70E-12	5.09E-07
			TNFRSF11B[b]		
11	rs4273881	8	TNFRSF11B[b]	7.33E-19	5.31E-14
			COLEC10[b]		
12	rs12375331	8	TNFRSF11B[b]	1.52E-07	1.68E-03
			COLEC10[b]		
13	rs7936582	11	PPP6R3[b]	3.18E-08	3.51E-04

			SAPS3[c]		
14	rs7995240	13	AKAP11[b]	3.96E-05	2.36E-02
			FABP3P2[c]		
15	rs12430303	13	FABP3P2[c]	8.30E-09	9.71E-05
			TNFSF11[b]		
16	rs34955778	16	NTAN1 [b]	1.42E-04	3.59E-02
			PDXDC1[c]		
17	rs724404	16	LOC727992[c]	3.22E-07	3.10E-03
			LOC728654[c]		
18	rs198542	17	CACNA1G[a]	5.88E-05	1.49E-02
19	rs8073599	17	PSMD12[c]	6.05E-06	4.37E-02
			LOC729822[c]		
20	rs2741856	17	SOST[a]	9.59E-08	7.86E-04
21	rs2423512	20	JAG1[a]	9.83E-04	8.36E-03
			FAT1P1[c]		
22	rs6040061	20	JAG1[a]	5.94E-09	1.19E-04

Column definition: SNP, single nucleotide polymorphisms; CHR, chromosome; *p*.value LS, summary statistic *p* value of LS BMD SNP; cFDR.LS, conditional false discovery rate of LS BMD when conditioned on BW.

[a] This gene is previously reported to be associated with both BMD and BW

[b] This gene is previously reported to be associated with BMD

[c] This gene has never been reported to be associated with LS BMD
