# Supplementary material for: Identification of PDXDC1 as a novel pleiotropic susceptibility locus shared between lumbar spine bone mineral density and birth weight
Source: J Mol Med (Berl). 2022 Mar 22;100(5):723–34. doi: 10.1007/s00109-021-02165-0 (PMC9110509; doi:10.1007/s00109-021-02165-0)
Supplement: Supplementary file 3 — Supplementary file3 (PDF 125 KB) [file 109_2021_2165_MOESM3_ESM.pdf]

Identification of Potential Novel Pleiotropic Susceptibility Variants Common to Lumbar Spine Bone Mineral Density and Birth Weight

Yu-Qian Song, Shi-Di Hu, Xu Lin, Xiang-He Meng, Xiao Wang, Yin-Hua Zhang, Cheng Peng, Rui Gong, Tao Xu, Tong Zhang, Chen-Zhong Li, Dao-Yan Pan, Jia-Yi Yang, Jonathan Greenbaum, Jie Shen*, Hong-Wen Deng*

*** Corresponding authors:**

Hong-wen Deng, Ph. D.

Tulane Center for Biomedical Informatics and Genomics, School of Medicine, Tulane University, New Orleans, LA 70112, USA

School of Basic Medical Science, Central South University, Changsha, Hunan 410013, P. R., China

Tel: 1 504-988-1310 E-mail: hdeng2@tulane.edu

Jie Shen, Ph. D.

Department of Endocrinology and Metabolism, The Third Affiliated Hospital of Southern Medical University, Guangzhou, China.

Department of Endocrinology and Metabolism, Shunde Hospital of Southern Medical University (The First People's Hospital of Shunde Foshan), Foshan, Guangdong, China

Email: sjiesy@smu.edu.cn

Journal name: Journal of Molecular Medicine

Online Resource 3. BW SNPs given LS BMD BW (cFDR<0.05)

	SNP	CHR	Nearby Gene	*p*.value BW	cFDR.BW
1	rs7549007	1	TCHHL1[c]	2.10E-05	2.92E-02
2	rs6673081	1	ZBTB7B[b]	1.20E-09	1.48E-05
3	rs2886070	1	ARHGEF2[c]	1.00E-06	4.88E-03
			SSR2[c]		
4	rs1044299	1	PAPPA2[c]	1.50E-05	2.79E-02
5	rs1415181	1	CENPF[c]	2.80E-06	1.06E-02
			LOC391162[c]		
6	rs6424243	1	LOC729336[c]	7.60E-06	1.62E-02
7	rs6700896	1	LEPR[a]	3.60E-05	4.97E-02
8	rs1488691	1	TGFBR3[c]	1.30E-05	1.55E-02
9	rs11690295	2	LOC100130388[c]	8.10E-06	7.37E-03
			MERTK[c]		
10	rs12623454	2	FLJ14816[c]	3.00E-05	2.73E-02
			GLI2[c]		
11	rs6437106	2	ACVR1C[c]	1.20E-05	1.95E-02
12	rs4675095	2	IRS1[a]	2.50E-05	3.77E-02
13	rs4233701	2	KLHL29[c]	3.10E-06	1.47E-02
14	rs2339940	2	C2orf44[c]	2.70E-06	7.27E-03
15	rs6735418	2	LOC100128048[c]	1.80E-05	3.06E-02

			ZFP36L2[c]		
16	rs11889485	2	PRKCE[c]	1.20E-13	6.88E-09
			EPAS1[b]		
17	rs7597315	2	PRKCE[c]	3.50E-10	5.83E-06
			EPAS1[b]		
18	rs6726089	2	EPAS1[b]	3.60E-06	1.29E-02
			LOC388946[c]		
19	rs3772587	3	CPB1[c]	6.70E-06	1.62E-02
20	rs9877642	3	LEKR1[b]	3.60E-05	2.40E-02
			CCNL1[b]		
21	rs2306531	3	LEKR1[b]	5.90E-06	7.81E-03
			CCNL1[b]		
22	rs2214409	4	LOC391679[c]	2.20E-05	3.68E-02
			TET2[c]		
23	rs12643660	4	PPA2[c]	3.50E-05	4.79E-02
24	rs2087826	4	HHIP[b]	3.00E-09	2.56E-05
25	rs740672	4	KIAA1276[c]	1.70E-08	1.02E-04
26	rs10008032	4	KLF3[c]	7.30E-05	4.88E-02
			TLR10[c]		
27	rs17015692	4	GPRIN3[c]	3.70E-05	3.12E-02
			SNCA[a]		

28	rs4704942	5	LOC100130177[c]	8.80E-07	5.61E-03
			EBF1[b]		
29	rs12656216	5	SKP2[c]	8.00E-06	1.17E-02
30	rs4699908	5	ACTBL2[c]	6.90E-06	1.43E-02
			LOC728103[c]		
31	rs16887484	5	ACTBL2[c]	2.40E-05	2.20E-02
			LOC728103[c]		
32	rs3756668	5	PIK3R1[c]	8.00E-06	9.05E-03
33	rs32902	5	CRHBP[b]	1.50E-05	2.69E-02
			AGGF1[c]		
34	rs1975427	5	LHFPL2[c]	1.40E-04	1.55E-02
35	rs12197879	6	CCDC170[c]	5.10E-03	2.55E-02
36	rs1293935	6	ESR1[a]	2.00E-10	2.80E-09
37	rs1361024	6	ESR1[a]	1.60E-05	1.35E-02
38	rs1389923	6	PDE10A[c]	1.90E-05	3.27E-02
			CCDC170[c]		
39	rs4712542	6	CDKAL1[b]	3.00E-05	4.58E-02
40	rs4144650	6	CDKAL1[b]	1.60E-07	1.14E-03
41	rs1547669	6	MLN[c]	6.70E-06	7.20E-03
			LOC100132252[c]		
42	rs11961715	6	GRM4[c]	2.30E-06	7.96E-03

43	rs6918981	6	LOC646766[c]	3.20E-06	1.39E-02
			NUDT3[c]		
44	rs1012635	6	CDKAL1[b]	1.00E-09	1.16E-05
45	rs1415701	6	L3MBTL3[b]	2.60E-09	2.82E-05
46	rs4143341	7	LOC100129824[c]	4.20E-06	1.24E-02
47	rs328890	7	DPY19L1[c]	3.40E-05	4.18E-02
48	rs2191883	7	TBX20[b]	5.00E-06	1.43E-02
49	rs12536475	7	TBX20[b]	1.10E-05	2.13E-02
			HERPUD2[c]		
50	rs1003573	7	CAMK2B[c]	4.60E-06	1.33E-02
51	rs17133917	7	GRB10[b]	1.50E-05	1.07E-02
52	rs7018178	8	SLC45A4[b]	5.70E-06	1.61E-02
			GPR20[c]		
53	rs732563	8	LOC646708[c]	7.20E-07	4.41E-03
			SLC25A37[c]		
54	rs328294	8	FGFR1[a]	6.20E-05	4.78E-02
			FLJ43582[c]		
55	rs2150052	9	LPAR1[b]	2.20E-08	1.18E-04
			OR2K2[c]		
56	rs2540074	9	STRBP[b]	7.40E-09	7.12E-05
57	rs9721852	9	UNQ6494[c]	6.30E-06	1.67E-02

			IL6RL1[c]		
58	rs10993068	9	PTPDC1[c]	7.20E-05	4.72E-02
			LOC158257[c]		
59	rs28536742	9	PTCH1[a]	1.50E-11	7.52E-08
			PSMA7P[c]		
60	rs1797081	10	CUBN[b]	2.80E-05	4.28E-02
61	rs17566087	10	HKDC1[c]	1.10E-05	2.39E-02
62	rs11187076	10	IDE[b]	1.40E-08	5.69E-05
			KIF11[c]		
63	rs6484487	11	MPPED2[c]	5.20E-06	1.49E-02
64	rs4980550	11	IFITM9P[c]	1.90E-04	4.89E-02
			CCND1[c]		
65	rs611003	11	IFITM9P[c]	1.80E-05	2.85E-02
			CCND1[c]		
66	rs6539026	12	C12orf48[c]	5.40E-05	4.90E-02
67	rs7309412	12	IGF1[a]	2.90E-06	1.32E-02
			LOC283432[c]		
68	rs6539049	12	IGF1[a]	1.10E-05	2.17E-02
			LOC283432[c]		
69	rs10774625	12	ATXN2[c]	2.70E-06	7.99E-03
70	rs895964	12	ITPR2[b]	2.30E-07	1.32E-03

71	rs11170457	12	ZNF740[c]	2.40E-05	3.52E-02
72	rs12306172	12	HOXC4[c]	3.70E-06	1.18E-02
			LOC644222[c]		
73	rs4762119	12	LOC204010[c]	1.40E-05	2.63E-02
			HMGA2[a]		
74	rs10878353	12	HMGA2[a]	2.60E-08	1.95E-04
			LOC729484[c]		
75	rs11175992	12	HMGA2[a]	2.60E-06	1.30E-02
			LOC729484[c]		
76	rs2013116	12	CLLU1[c]	6.50E-05	4.82E-02
			LOC100132126[c]		
77	rs7302663	12	CRADD[c]	1.70E-05	3.00E-02
78	rs1042725	12	HMGA2[a]	3.10E-32	2.71E-27
79	rs12865243	13	COG6[c]	4.20E-06	1.40E-02
			LOC646982[c]		
80	rs9568036	13	RB1[b]	5.70E-05	3.39E-02
81	rs1319046	15	FAM169B[c]	1.60E-05	2.64E-02
			IGF1R[a]		
82	rs34955778	16	NTAN1[c]	1.20E-03	1.60E-02
			PDXDC1[c]		
83	rs17767418	17	GOSR1[c]	2.10E-07	9.39E-04

84	rs17826255	17	LOC646021[c]	2.10E-05	3.41E-02
85	rs9303285	17	RARA[c]	1.40E-04	4.34E-02
86	rs198542	17	CACNA1G[a]	1.20E-03	1.74E-02
87	rs757558	17	AXIN2[c]	1.50E-05	2.81E-02
			CCDC46[c]		
88	rs9962540	18	CABLES1[c]	3.50E-06	1.20E-02
89	rs17384555	18	EPB41L3[c]	5.90E-06	1.45E-02
			LOC645355[c]		
90	rs10514019	18	LOC100132647[c]	1.90E-05	1.94E-02
			CBLN2[c]		
91	rs2423512	20	JAG1[a]	9.50E-08	9.41E-06
			FAT1P1[c]		
92	rs6057610	20	RP11-410N8.4[c]	4.30E-11	1.09E-06
			BCL2L7P1[c]		
93	rs6016377	20	HSPEP1[c]	9.50E-10	1.38E-05
			MAFB[a]		
94	rs6072263	20	TOP1[c]	1.80E-06	9.17E-03
95	rs2823025	21	NRIP1[a]	5.10E-07	3.17E-03
			CYCSP42[c]		
96	rs4823047	22	NIPSNAP1[c]	5.50E-05	4.91E-02
			THOC5[c]		

97	rs9611018	22	UNC84B[c]	6.20E-05	4.67E-02
			DNAL4[c]		
98	rs1548304	22	LOC388906[c]	2.10E-05	3.06E-02
			NFAM1[c]		
99	rs5765273	22	SMC1B[c]	3.90E-06	6.35E-03

Column definition: SNP, single nucleotide polymorphisms; CHR, chromosome; *p*.value BW, summary statistic *p* value of BW SNP; cFDR.BW, conditional false discovery rate of BW when conditioned on LS BMD.

[a]This gene is previously reported to be associated with both BW and BMD.

[b]This gene is previously reported to be associated with BW.

[c]This gene has never been reported to be associated with BW.
