# Supplementary material for: Identification of PDXDC1 as a novel pleiotropic susceptibility locus shared between lumbar spine bone mineral density and birth weight
Source: J Mol Med (Berl). 2022 Mar 22;100(5):723–34. doi: 10.1007/s00109-021-02165-0 (PMC9110509; doi:10.1007/s00109-021-02165-0)
Supplement: Supplementary file 4 — Supplementary file4 (PDF 77 KB) [file 109_2021_2165_MOESM4_ESM.pdf]

Identification of Potential Novel Pleiotropic Susceptibility Variants Common to Lumbar Spine Bone Mineral Density and Birth Weight

Yu-Qian Song, Shi-Di Hu, Xu Lin, Xiang-He Meng, Xiao Wang, Yin-Hua Zhang, Cheng Peng, Rui Gong, Tao Xu, Tong Zhang, Chen-Zhong Li, Dao-Yan Pan, Jia-Yi Yang, Jonathan Greenbaum, Jie Shen*, Hong-Wen Deng*

*** Corresponding authors:**

Hong-wen Deng, Ph. D.

Tulane Center for Biomedical Informatics and Genomics, School of Medicine, Tulane University, New Orleans, LA 70112, USA

School of Basic Medical Science, Central South University, Changsha, Hunan 410013, P. R., China

Tel: 1 504-988-1310 E-mail: hdeng2@tulane.edu

Jie Shen, Ph. D.

Department of Endocrinology and Metabolism, The Third Affiliated Hospital of Southern Medical University, Guangzhou, China.

Department of Endocrinology and Metabolism, Shunde Hospital of Southern Medical University (The First People's Hospital of Shunde Foshan), Foshan, Guangdong, China

Email: sjiesy@smu.edu.cn

Journal name: Journal of Molecular Medicine

Online Resource 4. Two-sample MR analysis

Outcome	Exposure	Method	nsnp	Beta (95%CI)	SE	Pval
LS BMD	BW	Inverse variance weight	46	-0.062 (-0.184,0.060)	0.062	0.320
		Maximum likelihood	46	-0.062 (-0.098,0.098)	0.050	0.214
