# Supplementary figures and images for: Identification of PDXDC1 as a novel pleiotropic susceptibility locus shared between lumbar spine bone mineral density and birth weight
Source: J Mol Med (Berl). 2022 Mar 22;100(5):723–34. doi: 10.1007/s00109-021-02165-0 (PMC9110509; doi:10.1007/s00109-021-02165-0)

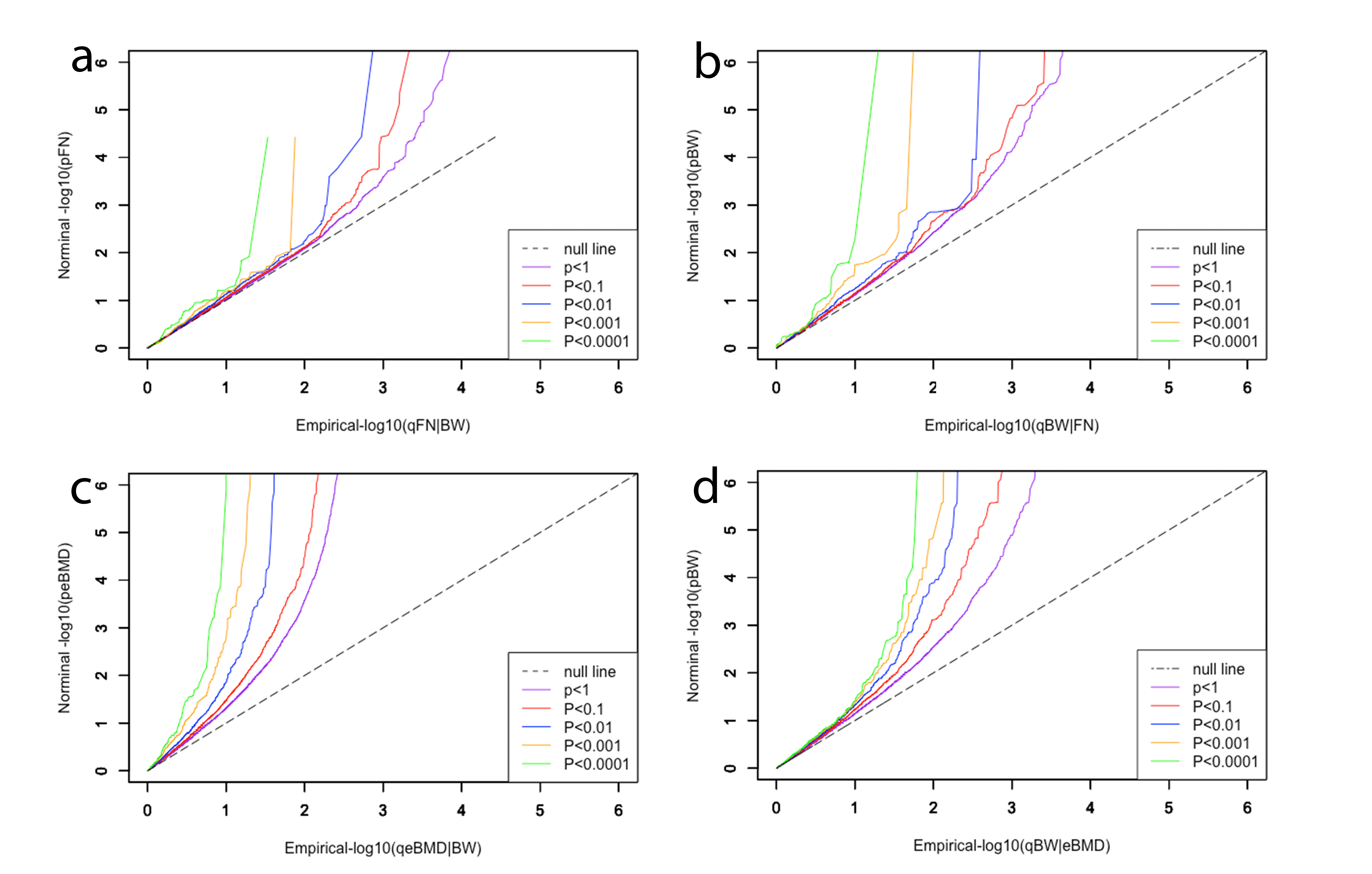

Supplement: Supplementary file 5 — Supplementary file5 (TIF 4643 KB) [file 109_2021_2165_MOESM5_ESM.tif]
